# Does previous standard percutaneous nephrolithotomy impair retrograde intrarenal surgery outcomes?

**DOI:** 10.1590/S1677-5538.IBJU.2021.0253

**Published:** 2021-05-04

**Authors:** Alexandre Danilovic, Fábio César Miranda Torricelli, Giovanni Scala Marchini, Carlos Batagello, Fabio Carvalho Vicentini, Olivier Traxer, Miguel Srougi, William C. Nahas, Eduardo Mazzucchi

**Affiliations:** 1 Faculdade de Medicina da Universidade de São Paulo Hospital das Clínicas Departamento de Urologia São PauloSP Brasil Departamento de Urologia, Hospital das Clínicas, Faculdade de Medicina da Universidade de São Paulo - FMUSP, São Paulo, SP, Brasil; 2 Sorbonne Université Hôpital Tenon Paris France Sorbonne Université, GRC n 20 Lithiase Renale, AP-HP, Hôpital Tenon, F-75020 Paris, France, University, Paris, France; 3 Faculdade de Medicina da Universidade de São Paulo Hospital das Clínicas Divisão de Urologia São PauloSP France Divisão de Urologia, Hospital das Clínicas, Faculdade de Medicina da Universidade de São Paulo, São Paulo, SP, Brasil

**Keywords:** Ureteroscopy, Kidney Calculi, Nephrolithotomy, Percutaneous

## Abstract

**Purpose:**

The objective of this study is to evaluate the impact of a previous standard percutaneous nephrolithotomy (PCNL) on the outcomes of retrograde intrarenal surgery (RIRS).

**Materials and Methods:**

Outcomes of RIRS performed from January 2017 to January 2020 in adult patients with residual stone fragments ≤20mm after a standard PCNL (Post-PCNL) and symptomatic adult patients with kidney stones ≤20mm (Control) were prospectively studied. Stone-free rate (SFR) was evaluated on a postoperative day 90 non-contrast computed tomography. Surgical complications based on Clavien-Dindo classification during the 90 days of follow-up were recorded.

**Results:**

Outcomes of 55 patients and 57 renal units of the post-PCNL group were compared to 92 patients and 115 renal units of the control group. SFR was lower in post-PCNL group than in control (28/57, 49.1% vs. 86/115, 74.8%, p <0.001). Overall complications were more frequent in post-PCNL group (p=0.004). Infundibula strictures were identified and incised with laser in 15/57 (26.3%) renal units of the post-PCNL group. Thirteen renal units had infundibulum stricture at the site of previous percutaneous tract (13/15; 86.7%, p=0.004) and one renal unit had three infundibula strictures. Postoperative complications were not affected by the treatment of infundibula strictures (p=0.198).

**Conclusions:**

Previous standard PCNL significantly impairs the outcomes of RIRS. Infundibula strictures can be found in 26.3% of the patients with residual stone fragments after standard PCNL for large burden kidney stones. The main site of infundibulum stricture after standard PCNL is the infundibulum of the entry ca*lyx*.

## INTRODUCTION

Percutaneous nephrolithotomy (PCNL) is the first-line therapy for large kidney stones (1-3). Recent technical improvements decreased complications and increased stone-free rate (SFR) of PCNL (4, 5). However, treatment of a large stone burden is time consuming and usually staghorn kidney stones are still managed by standard PCNL (6, 7). Despite all efforts, some residual fragments may persist after PCNL and need to be addressed to avoid re-growth or ureteral obstruction (8, 9).

Retrograde intrarenal surgery (RIRS) is an appealing choice for the treatment of these residual fragments (10, 11). Multiple fragments can be treated simultaneously and, at least theoretically, the entire collecting system may be accessed by flexible ureteroscopy (12, 13). SFR of RIRS for kidney stones ≤20mm ranges from 55% to 75% measured by non-contrast computed tomography (NCCT) (14, 15). However, there are few studies looking at the outcomes of RIRS after a previous PCNL (10, 11).

There are few data describing abnormalities of the renal collecting system anatomy after PCNL. It was already demonstrated in both porcine and cadaveric model that dilation tracts up to 24Fr had significantly smaller parenchymal fissures and reduced capsule rupture than 30Fr tracts (16). Other authors previously reported 2% asymptomatic infundibula strictures after standard PCNL (17). During PCNL, navigation with rigid nephroscope is frequently required to reach a stone in a different calyx than the entry calyx. A steep angle <75° between the entry calyx of percutaneous tract and the calyx with stone prevents rigid nephroscopy navigation (18). Renal scar may occur in up to 48% of the site of percutaneous access (19). Our hypothesis was that dilation of the infundibulum with a large bore tract during standard PCNL, mainly in large stone burden, cause stricture at some point of the renal collecting system, particularly at the entry calyx of the percutaneous access, and cause difficulty to reach a fragment during RIRS for residual stone fragments after PCNL. The aim of this study was to evaluate the impact of a previous standard PCNL on the outcomes of RIRS.

## PATIENTS AND METHODS

### Study design

Adult patients with residual stone fragments up to 20mm after a standard PCNL (Group: Post-PCNL) and symptomatic adult patients with kidney stones up to 20mm (Group: Control) without any previous kidney or ureteral surgery submitted to RIRS in our Institution from January 2017 to January 2020 were prospectively studied. Patients with kidney malformations, urinary diversion, pregnancy, those submitted to previous kidney or ureteral surgery or combined surgery concomitant to RIRS such as PCNL or transurethral resection of prostate (TURP), or failure to insert ureteral access sheath (UAS) were excluded from this study. The Institutional ethics committee approved the study protocol (IRB No. 11851) and written informed consent was obtained from all patients according to the Declaration of Helsinki Ethical Principles for Medical Research involving Human Subjects.

Stone burden before PCNL was classified using the Guy’s grading system using NCCT for all patients (20). Standard PCNL by experienced surgeons were performed in supine or prone position according to surgeon’s preference under fluoroscopy and ultrasound guidance. Dilation was accomplished using fascia dilators to fit a 30Fr Amplatz sheath and a 26Fr rigid nephroscope was used in all cases. Stone burden, patient position, calyx of access, number of tracts and use of nephrostomy tube were recorded in every case.

NCCT were performed during the week before of RIRS with no stent in place for all patients to look for stone burden using a 64-slice GE Lightspeed CT Scanner^®^ (General Electrics, USA) and slice thickness of 1mm. Stone features were evaluated in the magnified (400%) bone window (width, 1600UH/level, 500UH) in the three axes. Stone size sum was considered the sum of the longest diameter of each stone in the renal unit. Stone volume sum was calculated using the sum of the volume of each stone in the renal unit using ellipsoid formula as 0.167x π x length x width x depth (21, 22) Stone density was measured by free hand region of interest (ROI) determination coincident with the stone borders (23). Infundibulopelvic angle of the inferior calyx was measured in all patients using the method previously reported (24).

Standardized RIRS was performed under general anesthesia at least six weeks after PCNL. A Nitinol 0.035” guide wire (Coloplast, DK) and a PTFE 0.035” guide wire were inserted up to the renal pelvis under tactile control. An ureteral access sheath (UAS) 10/12Fr x 35cm (Coloplast, DK) was placed up to the upper ureter in all cases and a flexible ureteroscope (URF-P5^®^, Olympus, JN) was inserted for direct inspection of all renal calices before lithotripsy. Laser lithotripsy was performed with a 270-micron Holmium laser fiber (Dornier, USA) using 12-18Hz and 0.4-0.6 J laser settings. Stone fragments >2mm were removed with a 1.5Fr tipless basket (Coloplast, DK). Pyelography through the UAS was performed at the end of procedures and a 6Fr silicone double J stent (Coloplast, DK) was located. The UAS was removed under direct ureteroscopic vision and inspected looking for ureteral lesions according to the Post-Ureteroscopic Lesion Scale (PULS) (25). Operative time was defined from the beginning of cystoscopy till the end of double J insertion for each renal unit. Patients were discharged on the same day except if visual analogic scale (VAS) for pain was >3. Patients were maintained with standardized oral analgesics until removal of the double J stent on postoperative day (POD) (10).

Stone-free rate after RIRS was also evaluated on a POD 90 NCCT by a senior radiologist, blinded for the groups, for each renal unit. We considered stone-free when no fragments were found. Surgical complications based on Clavien-Dindo classification during the 90 days of follow-up were recorded (26).

### Statistical Analysis

Categorical data were reported as frequency and percentage and continuous data as mean and standard deviation. Continuous variables were compared using ANOVA or the Student’s t test for independent groups, whereas categorical variables were compared using the Chi-square and Fisher’s exact test. SPSS^®^ Statistics Version 20 (IBM Corp©, USA) was used for statistical analysis. Sample size of 57 renal units for residual fragments group and 115 renal units for control group (case-control study 1:2 proportion) was calculated to a test power of 90% and alpha error of 0.05 assuming SFR of 50% for residual fragments group and 75% for control group (15).

## RESULTS

**Figure 1:**
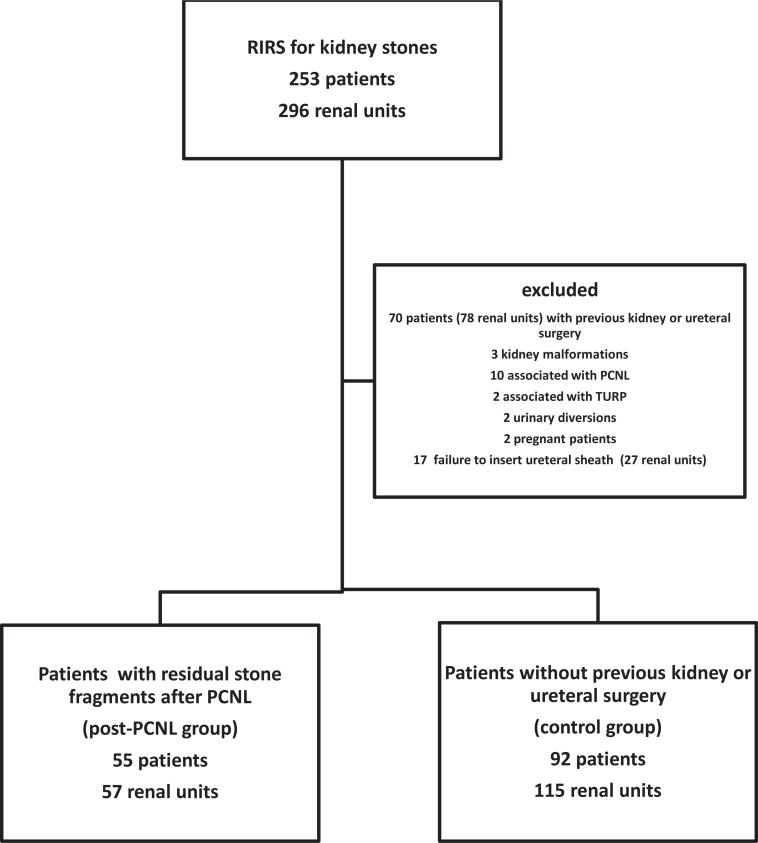
Flow diagram of the enrollment of participants in the study.

Outcomes of 55 patients and 57 renal units of the RIRS post-PCNL group were compared to 92 patients and 115 renal units of the RIRS control group (Figure-1). During the same period of time, a total of 397 PCNL were performed in our Institution. Stone burden before PCNL was Guy´s grade 1 in 2/57 renal units (3.5%); grade 2 in 1/57 (1.8%); grade 3 in 24/57 (42.1%); and grade 4 in 30/57 (52.6%). Standard PCNL was performed in supine position in 47/57 (82.5%) of the cases. Number of percutaneous tracts was one in 38/57 (66.7%) and two in 19/57 (33.3%). Primary access was obtained in the lower calyx in 32/57 (56.1%); middle calyx in 19/57 (33.3%); and upper calyx in 6/57 (10.5%). Secondary access was obtained in the lower calyx in 8/19 (42.1%); middle calyx in 5/19 (26.3%); and upper calyx in 6/19 (31.6%). Flexible nephroscopy was done in 24.6% at the end of PCNL. Nephrostomy tube was placed at the end of the procedures in 48/57 (84.2%) cases.

Clinical and stone data comparing post-PCNL and control group are depicted in Table-1. Sex, age, BMI and Charlson comorbidity index were similar between groups (27). Both groups had similar stone size and volume sum, stone location and infundibulopelvic angle of the inferior calyx.

**Table 1:** Clinical features of post PCNL RIRS vs. Control.

Feature			Post-PCNL RIRS	Control	p
Female, N (%)			36 (65.5%)	60 (65.2%)	1
Age (mean ± SD), years			47.4 ± 12.9	46.7 ± 14.1	0.759
BMI (mean ± SD), Kg/m^2^			28.7 ± 5.7	28.0 ± 4.8	0.47
**Charlson, N (%)**					**0.808**
	0	19 (34.5%)	36 (39.1%)	
	1	9 (16.4%)	18 (19.6%)	
	2	12 (21.8%)	17 (18.5%)	
	3	4 (7.3%)	7 (7.6%)	
	4	7 (12.7%)	7 (7.6%)	
	5	2 (3.6%)	2 (2.2%)	
	6	2 (3.6%)	1 (1.1%)	
	7	0	2 (2.2%)	
	8	0	1 (1.1%)	
	9	0	0	
	10	0	1 (1.1%)	
Stone side, Right N (%)			29 (50.9%)	62 (53.9%)	0.707
Bilateral, N (%)			2 (3.6%)	23 (25%)	**<0.001**
Stone size sum (mean ± SD), mm			13.47 ± 5.21	14.92 ± 7.26	0.137
Stone volume sum (mean ± SD), mm^3^			343.8 ± 340.3	436.4 ± 473.7	0.145
Stone density (mean ± SD), HU			749.3 ± 269.3	989.3 ± 330.2	**<0.001**
**Stone location**					
Superior calyx, N (%)			19 (33.1%)	53 (46.1%)	0.140
		Middle calyx, N (%)	22 (38.6%)	58 (50.4%)	0.149
		Inferior calyx, N (%)	36 (63.2%)	78 (67.8%)	0.608
		Pelvis, N (%)	6 (10.5%)	24 (20.9%)	0.134
		Infundibulopelvic angle of the inferior calyx ≤ 40°, N (%)	24 (42.1%)	66 (57.4%)	0.074
		Stone size inferior calyx (mean ± SD), mm	8.93 ± 4.32	8.52 ± 14.05	0.541
**Stone composition**					**<0.001**
		Calcium oxalate monohydrate, N (%)	12 (21.1%)	49 (42.6%)	
		Calcium oxalate dihydrate, N (%)	11 (19.3%)	56 (48.7%)	
		Calcium phosphate, N (%)	6 (10.5%)	10 (8.7%)	
		Uric Acid, N (%)	5 (8.8%)	0	
		Struvite, N (%)	23 (40.4%)	0	

Outcomes of post-PCNL were compared to control in Table-2. Stone-free rate was lower in post-PCNL group than in control (28/57, 49.1% vs. 86/115, 74.8%, p <0.001, respectively). Operative time was longer in the post-PCNL group (p <0.001) and length of hospital stay was similar between groups (p=0.346).

**Table 2:** Outcomes of post-PCNL RIRS vs. Control.

Outcome		Post-PCNL RIRS	Control	p
Operative time (mean ± SD), min.		85.60 ± 31.82	54.48 ± 26.73	<0.001
Hospitalization time (mean ± SD), h		12.44 ± 2.26	14.09 ± 16.46	0.346
**Residual stone fragment rate, N (%)**				
	0 mm	28 (49.1%)	86 (74.8%)	<0.001
	0 – 2 mm	9 (15.8%)	9 (7.8%)	0.119
	> 2 mm	20 (35.1%)	20 (17.4%)	0.013
**PULS**				**0.022**
	0	46 (80.7%)	108 (93.9%)	
	1	7 (12.3%)	5 (4.3%)	
	2	4 (7.0%)	1 (0.9%)	
	3	0	1 (0.9%)	
**Clavien-Dindo classification**				**0.004**
	0	42 (73.7%)	95 (82.6%)	
	I	13 (22.8%)	14 (12.2%)	
	II	1 (1.8%)	5 (4.3%)	
	IIIb	0	1 (0.9%)	
ER visits, N (%)		3 (5.5%)	16 (17.4%)	0.043

**PULS** = post-ureteroscopic lesion scale; ER = emergency room

Although ureteral lesions were more frequent in post-PCNL (p=0.022), the only PULS 3 occurred in the control group. Similarly, although overall complications were more frequent in post-PCNL group (p=0.004), the only Clavien-Dindo IIIb occurred in the control group. Emergency room (ER) visits were also more frequent in the control group (3/55, 5.5% vs. 16/92, 17.4%, p=0.043). Patients from control group visited ER due to urinary infection (two patients), pain (one patient four times and five patients twice) and one patient for ureteral stent placement. Three patients from the post-PCNL group visited the ER due to urinary infection, pain and urinary infection plus pain one each.

Infundibula strictures were identified and incised with laser in 15/57 (26.3%) renal units of the post-PCNL group and none in control group. Thirteen renal units had infundibulum stricture at the site of previous percutaneous tract (13/15; 86.7%, p=0.004) and one renal unit had three infundibula strictures. Finding of infundibulum stricture during RIRS was not associated with inferior calyx percutaneous tract of previous PCNL (p=0.772) or with flexible nephroscopy use at the end of PCNL (p=0.569). Postoperative complications were not affected by the treatment of infundibula strictures (p=0.198). The presence of infundibulum stricture did not affect SFR in post-PCNL RIRS (p=0.261). However, residual stone fragments after post-PCNL RIRS were located in the same calyx of infundibulum stricture in 7/10 (70%). The finding of infundibulum stricture during RIRS was not correlated to time in between PCNL and RIRS (r=12, p=0.359). Also, the mean of time in days in between PCNL and RIRS was similar comparing patients with and without infundibulum stricture (128.8±107.4 vs. 103.0±88.4, p=0.373).

## DISCUSSION

This prospective study demonstrated lower SFR for RIRS after standard PCNL compared to RIRS performed in patients without any previous ureteral or kidney surgery using NCCT as imaging control exam for all patients. Overall complications were higher in post-PCNL group albeit the only major complication (Clavien IIIb) occurred in a patient from the control group. Infundibula strictures were found in 26.3% of the patients with residual stone fragments after standard PCNL for large burden kidney stones. The main site of infundibulum stricture after standard PCNL was the infundibulum of the entry calyx.

Our hypothesis was that previous standard PCNL could cause distortion of the collecting system anatomy and difficult RIRS. Large bore percutaneous tract in a normal albeit narrow infundibulum and/or steep torque to reach a kidney stone in other calyx might result in the formation of scar tissue and stricture. The vast majority of the cases of the post-PCNL group had a PCLN due to large stone burden (94.7% Guy’s III or IV) and was operated in supine position (82.5%). None of the patients required three or more tracts during PCNL. Those are challenging PCNL requiring longer operations and are susceptible to steeper torque. In this context we found among these patients fifteen infundibulum strictures that were treated with laser incision in 57 renal units (26.3%). An inferior calyx was the preferred primary access for PCNL in this study (56.1%) and also the preferred secondary access (42.1%). Thirteen renal units had one infundibulum stricture at the site of previous percutaneous tract and one renal unit had three infundibula strictures. The majority of infundibula strictures occurred at the infundibulum of the entry calyx (86.7%, p=0.004). We used the same technique to cut infundibulum stricture as we use to incise calyceal diverticulum neck (28). However, laser cutting of an infundibulum stricture is a more challenging procedure when compared to the opening of a calyceal diverticulum neck because those strictures can be long and lobar arteries can be very close to the laser zone. This could justify the longer operative time of post-PCNL group (p <0.001). Even though, no postoperative complications were associated to infundibulum stricture treatment (p=0.198).

During the same period of time of this study, 397 PCNL were performed in our Institution. Considering all PCNL performed, the total diagnosed infundibulum stricture rate would be 2.3%, similar to a previous retrospective study by Parsons et al. that reported five asymptomatic infundibula strictures after 223 PCNL (2%) (17). Possibly, the reported low number of infundibula strictures after PCNL was because of the retrospective nature of that study and the fact that the patients are usually asymptomatic, like in our series. However, the 26.3% rate of infundibulum stricture of our study reflects the incidence of the particular group of patients submitted to standard 30Fr PCNL due to a large stone burden with residual stone fragments. This is another evidence to support the systematic image study after PCNL.

Other authors also reported inferior results of RIRS when used as second-line therapy after shock wave lithotripsy (SWL) or PCNL compared to RIRS as first-line therapy (29). In a retrospective study, authors enrolled 51 patients in the second-line therapy group, only eight cases after PCNL, and compared to 42 patients submitted to RIRS as first-line therapy. Stone-free rate evaluated by US and KUB was lower in the second-line therapy group after 6 weeks (80% vs. 67%). The authors speculated some anatomical unfavorable aspects for SWL might apply to RIRS and suggest proper assessment of the infundibulum anatomy, particularly in the lower pole. In a previous publication, we also noted the importance of the infundibulopelvic angle and showed a simple method to measure the angle on NCCT (24). In the present study, both post-PCNL RIRS and control RIRS groups had similar rate of steep lower pole infundibulopelvic angle. Although the mere presence of infundibulum stricture did not affect SFR in post-PCNL RIRS (p=0.261), as much as 70% of the residual stone fragments after post-PCNL RIRS of the patients with infundibula strictures were located in the same calyx of the infundibulum stricture.

Post-PCNL RIRS is a safe procedure. All complications were Clavien I or II. Overall complications classified by Clavien-Dindo were more frequent in post-PCNL group (p=0.004) possibly, due to longer operative time (p <0.001). However, the only one Clavien-Dindo IIIb occurred in the control group. Emergency room (ER) visits were more frequent in the control group (16/92, 17.4% vs. 3/55, 5.5%, p=0.043). ER visits are a reliable comparison of how troublesome RIRS could be for a patient never managed before by endoscopy versus a patient previously submitted to PCNL. The vast majority of the patients from the control group that visited ER did so due to pain and patients from post-PCNL that visited ER did so due to urinary infection. Ureteral lesions were more frequent in post-PCNL (p=0.022), but the only one PULS 3 occurred in the control group.

Our study has some limitations. Although it was a prospective comparative study, it was conducted in a single Institution and other similar studies in different Institutions are important for the external validation of our results. Almost all patients from post-PCNL group were submitted to standard large bore PCNL due to complex kidney stones. This population may differ from other institution’s patients and the frequency of infundibulum strictures may vary. Patients of post-PCNL RIRS group had more struvite stones and consequently lower density than patients of control group. However, no impact was noted in urinary tract infection rate after RIRS or in operative time of RIRS. Perhaps, miniaturized PCNL could reduce the incidence of infundibula strictures and improve SFR of post-PCNL RIRS. Also, we used only one type of flexible ureteroscope (URF-P5^®^, Olympus, JN) in this study. This flexible ureteroscope has deficiencies to deal with right side lower pole kidney stones (30). Although both groups were similar regarding stone side for treatment by RIRS, if a flexible ureteroscope with working channel in 3 o’clock position was available, SFR might be improved.

## CONCLUSIONS

Previous standard PCNL significantly impairs the outcomes of RIRS. Infundibula strictures can be found in 26.3% of the patients with residual stone fragments after standard PCNL for large burden kidney stones. The main site of infundibulum stricture after standard PCNL is the infundibulum of the entry calyx.

## ABBREVIATIONS

PCNL: percutaneous nephrolithotomy
RIRS: retrograde intrarenal surgery
TURP: transurethral resection of the prostate
N: number
SD: standard deviation
Kg: kilogram
M: meter
Mm: milliliter
HU: Hounsfield Units
Min: minute
H: hour
